# Production of virulence factors by species of *Candida albicans *isolated from urine culture

**DOI:** 10.1186/1753-6561-8-S4-P19

**Published:** 2014-10-01

**Authors:** Laura Wiebusch, Danielle Lonchiati, Luana Rodrigues, Allan Rodrigues, Adriana Almeida, Kelly Oliveira

**Affiliations:** 1 Universidade Federal da Grande Dourados, Dourados, Brazil

## Background

The candiduria can be defined as the observation of yeasts in urine examination [1]. The ability of yeast to adhere, to infect and cause the disease in the set is defined as a potential virulence or pathogenicity [2]. Virulence factors expressed by Candida species may vary depending on the type of infection, the location and the stage of the infection, and the nature of the host response. The production of proteases and phospholipases enzymes and biofilm production of some virulence factors are expressed by the Candida species that degrade host tissues [3]. Thus, this study aimed to evaluate the production of proteinase, phospholipase and biofilms of Candida species isolated from patients at the university hospital in Dourados - MS.

## Methods

In this study we used samples of *Candida albicans *from samples of urine cultures of patients admitted to the University Hospital of Dourados, from June 2010 to June 2012. The yeasts were isolated and identified according to the conventional method. The phospholipase production was verified by the method of egg yolk agar plate, the proteinase production by the method of agar containing bovine serum albumin by the method of biofilm and adherence to polystyrene. Tests to evaluate the enzymatic activity were performed in duplicate in three different times. During the study period, 24 yeasts were obtained from patients hospitalized.

## Results and conclusions

The samples analyzed in the phospholipase test, two (8.33%) were moderate with enzymatic activity and 23 (91.66%) showed no enzymatic activity. Thet proteinase test, 15 (50%) samples showed strong enzymatic activity. Seven samples (41.66%) showed moderate enzyme activity and two (12.5%) samples showed no enzymatic activity. The biofilm test, 22 (91.66%) samples showed biofilm formation and two (8.33%) did not showed biofilm formation. As shown, most of the samples showed no enzymatic activity in phospholipase production, but most isolates showed proteinase production and biofilm indicating the high virulence of these isolates. The values found, refers to the importance of the studies since the action of these enzymes in the host organism.
